# The Role of *Lysobacter antibioticus* HS124 on the Control of Fall Webworm (*Hyphantria cunea* Drury) and Growth Promotion of Canadian Poplar (*Populus canadensis* Moench) at Saemangeum Reclaimed Land in Korea

**DOI:** 10.3390/microorganisms9081580

**Published:** 2021-07-25

**Authors:** Jae-Hyun Moon, Sang-Jae Won, Chaw Ei Htwe Maung, Jae-Hyeok Choi, Su-In Choi, Henry B. Ajuna, Young Sang Ahn, Yong Hun Jo

**Affiliations:** 1 Department of Forest Resources, College of Agriculture and Life Sciences, Chonnam National University, Gwangju 61186, Korea; mjh132577@naver.com (J.-H.M.); lazyno@naver.com (S.-J.W.); cjh960728@gmail.com (J.-H.C.); suin917@naver.com (S.-I.C.); ajunahenry@mmu.ac.ug (H.B.A.); 2 Division of Agricultural and Biological Chemistry, Institute of Environmentally Friendly Agriculture, College of Agriculture and Life Sciences, Chonnam National University, Gwangju 61186, Korea; chaweihtwemaung@gmail.com; 3 Department of Applied Biology, Institute of Environmentally Friendly Agriculture, College of Agriculture and Life Sciences, Chonnam National University, Gwangju 61186, Korea

**Keywords:** entomopathogenic bacteria, cuticle degrading enzymes, forest pests, auxin, plant development, poplar biomass

## Abstract

*Populus canadensis* Moench forests established in Saemangeum-reclaimed land have been invaded by *Hyphantria cunea* Drury, causing defoliation and stunted growth. This study investigated the biocontrol potential of cuticle degrading chitinase and protease secreted by *Lysobacter antibioticus* HS124 against *H. cunea* larvae. In addition, *L. antibioticus* HS124 was examined for indole-3-acetic acid phytohormone production for plant growth promotion. To determine the larvicidal activity in the laboratory experiments, crude enzymes, bacteria culture, CY medium, and water (control) were sprayed on the larvae reared on natural diet in insect rearing dishes. Treatment with crude enzymes and bacteria culture caused 76.7% and 66.7% larvae mortality, respectively. The larvae cuticle, mainly composed of chitin and proteins, was degraded by cuticle-degrading enzymes, chitinase, and protease in both the bacteria culture and crude enzyme treatments, causing swelling and disintegration of the cuticle. Field application of the bacteria culture was achieved by vehicle-mounted sprayer. Bacterial treatment caused morphological damage on the larvae cuticles and subsequent mortality. Foliar application of the bacteria culture reduced tree defoliation by *H. cunea* and enhanced growth compared to the control. Especially, *L. antibioticus* HS124 produced auxins, and increased growth of poplar trees.

## 1. Introduction

**Figure 1:**
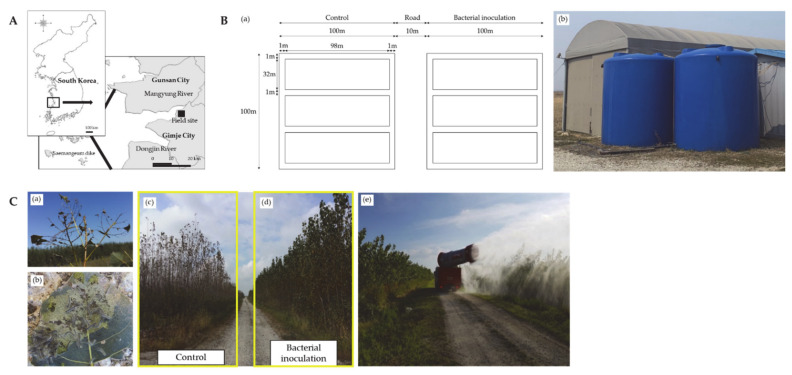
Locations of Saemangeum-reclaimed land (**A**). Illustration of field experimental site with the control and bacterial inoculation treatment plots (**B**a) and on-farm large-scale cultivation of *Lysobacter antibioticus* HS124 in fermentation tank (**B**b). Tree defoliation by *Hyphantria cunea* larvae in the control group (**C**a–**C**c) and *Populus canadensis* vegetation in the treatment group and foliar application of bacterial using a vehicle-mounted sprayer (**C**d–**C**e).

Global demand to produce fast-growing forest trees has progressively increased in recent years because of the impact on global climate and concerns about energy security [1,2]. Plantations of woody species with fast growth rates and short cultivation periods are a desirable source of renewable energy [3]. *Populus*, or poplars, are a genus of deciduous trees in the family Salicaceae with more than 30 species grown in Asia, Europe, Africa, and America [4,5]. These species are well known for their adaptability to unfavorable environments, including low fertility and high saline soils [4,5,6]. Among the *Populus* species, *Populus canadensis* Moench, a natural hybrid of *Populus deltoides* Marsh and *Populus nigra* var. *italica,* is a key species in forest tree plantations in temperate regions, accounting for over 90% of cultivated poplars globally [4]. In the late 19th century, the Korean government constructed Saemangeum-reclaimed land to increase the cultivatable cropland, in response to limited agricultural plains [7,8,9]. However, due to high soil salinity variations, salt tolerant species such as *P. canadensis* have been established for bioenergy production and carbon sequestration [9,10]. However, *P. canadensis* are highly vulnerable to fall webworm (*Hyphantria cunea* Drury) invasion (Figure 1C). The larvae of *H. cunea* are gregarious polyphagous pests that cause extensive forest defoliation and form innumerable webbings, which reduce the photosynthetic efficiency, leading to stunted growth and subsequent decrease in biomass production. *H. cunea* (Lepidoptera: Erebidae) is a native of North America, from where it accidentally spread to various parts of Europe and Asia [11]. The global warming phenomenon has significantly increased the density of *H. cunea,* which occurs mainly in warmer regions [12,13,14]. These larvae are known to infest and feed a broad host range with over 600 plant species including forest trees, fruit trees, and herbaceous and agricultural crops from late summer to early fall [11]. Even though trees can survive extensive defoliation, the loss of leaves reduces the rate of photosynthesis and leads to stunted tree growth [5]. Moreover, severe defoliation increases susceptibility and transmissibility of phytopathogens, resulting in tree mortality, longer rotation periods, and significant economic losses in commercial tree production [5].

Generally, chemical pesticides are extensively used to reduce pest invasions due to quick and consistent action. However, frequent use of synthetic pesticides poses serious problems, such as pesticide resistance, alteration in soil microbial diversity, and contamination of water, air, and soil ecosystem [15,16,17], in forest management. In addition, only about 0.1% of the applied pesticides reach the targeted organisms, while the remaining bulk escapes to the environment and adversely affects non-target beneficial organisms such as natural enemies, pollinators, and earthworms [15,16,17]. Thus, many researchers and non-governmental organizations have expressed concerns about environmental pollution from leached chemicals in the Saemangeum-reclaimed land. Consequently, the Korean government enacted a law prohibiting the use of chemical products in the Saemangeum-reclaimed land [7,8]. Therefore, the Korean forest services (KFS) cannot use pesticides in Saemangeum-reclaimed land, and there is a need to develop environmentally friendly techniques for controlling *H. cunea* and increase biomass production of *P. canadensis* plantations. Several pest management strategies, including the use of larvicidal plant extracts, sex pheromones, entomopathogens, and natural enemies, such as predators and parasitoids, have been employed in the management of various pests [11,18]. The use of natural enemies plays a major role in maintaining the pest population below the threshold level, but appropriate technologies for mass rearing and strategic release are still challenging [11,19]. The application of entomopathogenic bacteria, also known as biocontrol agents, has become increasingly attractive as eco-friendly alternatives to chemical pesticides [18,20,21]. Entomopathogenic bacteria have high specificity to target pests and are not harmful to humans, wild animals, and other organisms, including natural enemies [22]. However, the application of biocontrol agents on large-scale forest areas has been challenged by unfavorable environmental conditions (such as cold weather and dry conditions) which reduce the performance of entomopathogens and lower production efficiency [11,21].

The most widely used microbial pesticides are based on entomopathogenic bacterium, *Bacillus thuringiensis* (Bt) [20]. However, the long-term use of Bt as a classical biological control strategy has resulted in Bt toxin resistance in various orders of insect pests, including Lepidoptera, Coleoptera, and Diptera [23]. Therefore, there is an urgent need to develop additional microbial agents to control the growing pest population. Entomopathogenic bacteria interfere with the growth and survival of the insect pest through the production of diverse active metabolites, including toxins and cuticle degrading enzymes [18,20]. In particular, cuticle-degrading enzymes, such as chitinases and proteases produced by entomopathogenic bacteria, play a key role in breaking down the cuticles of insect pests, which are mainly composed of chitin and protein [20,21,24,25]. Cuticular chitin and cuticular protein are vital components of insect cuticles, but are also an easy target in insect pest management [25,26,27]. Chitinases are among the most valuable hydrolytic enzymes deployed by entomopathogenic bacteria to degrade vital structures of insect pests, such as the cuticle in the exoskeleton [28]. In addition, proteases have hydrolytic activity on various proteins present in the larvae cuticle which could cause decomposition of the insect pest exoskeleton [26,29]. Decomposed cuticles cause protective barriers to rupture which consequently leads to insect mortality [21]. Additionally, some bacteria play a vital role as biofertilizers, providing plants with phytohormones, such as auxin, which promote plant growth [7,8,30,31,32]. Phytohormone auxin improves plant growth by stimulating a wide range of processes, including cell division, tissue growth, and lateral root initiation [30,33]. Specifically, *Lysobacter antibioticus* strains have been reported to exhibit a pronounced inhibitory effect against insect pests [34] and the potential to promote biomass production through auxin production [31]. However, the simultaneous pesticidal effects and the promotion of biomass yield in bioenergy-producing trees has not been reported under field conditions. The cultivation of *P. canadensis* on Saemangeum-reclaimed land in Korea is constrained by limited knowledge regarding the management of *H. cunea* using ecofriendly methods. Therefore, the objective of this study was to investigate the biocontrol potential of *L. antibioticus* HS124 against the *H. cunea* larvae and increasing biomass yield of *P. canadensis* in the Saemangeum-reclaimed land. Treatment with entomopathogenic bacteria, *L. antibioticus* HS124, caused larvae mortality, prevented defoliation, and improved the growth of *P. canadensis* and can thus serve as an alternative to chemical use for simultaneous control of *H. cunea* larvae and for enhancing biomass production in the field.

## 2. Materials and Methods

### 2.1. Study Area Description

The study was conducted in Saemangeum-reclaimed land, in an estuary tidal flat that lays at the intersection of the Mangyung and Dongjin Rivers in the southwest coastal region of Korea (Figure 1A). This area is approximately 40,000 ha and is therefore one of the largest land reclamation projects in Korean history. The reclaimed land was not provided with landfill for soil improvements. The soils in the study area were mainly fluvial-marine deposits and the dominant soil is sandy loam, with a slope of 0 to 2%. High salinity and low soil fertility are the primary constraints limiting plant growth and land development in this region [6,7,8,9,10]. Reeds (*Phragmites communis* Trinius) are the dominant vegetation, while most woody plants barely grow in the area due to salt stress. The study area has a temperate climate with an annual mean temperature of 13.0 °C [14]. The mean annual precipitation on-site is about 1233.4 mm, with approximately 51.3% falling between June and August over the last 10 years [14].

Since 2012, the KFS has established 180 ha of *P. canadensis* plantations in the study area to produce wood pellets for bioenergy production. The experimental site (35°52′14″ N latitude, 126°46′2″ E longitude) was approximately 2 ha, planted at a density of 8000 cuttings per hectare (at a spacing of 1.25 m × 1.25 m). The experimental site was divided into two blocks of 1 ha each with a roadway of 10 m width running in between (Figure 1B). Each block was divided into 3 separate plots (98 m × 32 m each) and the plots were separated by a water channel of 1 m width and 1 m deep. (1) One block containing three plots (replicates) was allocated for *L. antibioticus* HS124 culture treatment and (2) the other block containing three replicates was assigned to the control group (without water and bacterial culture). The cuttings (approximately 100 cm high and 1 cm in diameter) were plant in the experiment plots in 2013. By 2015, the established trees were approximately 4.0 m high, with a root collar diameter of 4 cm, distributed uniformly in the experiment plots. However, in 2015, severe infestation by *H. cunea* larvae caused extensive defoliation of *P. canadensis* trees, creating innumerable webbed nests over the canopy, which reduced photosynthetic efficiency and subsequently decreased biomass yields (Figure 1). 

### 2.2. Bacterial Culture and Growth Conditions

The bacterial strain *L. antibioticus* HS124 was isolated from pepper field soils in Naju City, Korea [34]. Then, *L. antibioticus* HS124 was streaked on casitone-yeast extract agar (CYA) medium (bacto-casitone 3 g, CaCl_2_∙2H_2_O 1.36 g, yeast extract 1 g and agar 20 g in 1 L distilled water) and inoculated at 30 °C for 3 days. To examine growth conditions and the cuticle-degrading enzyme (chitinase and protease) activity of *L. antibioticus* HS124, 10 colonies were pre-inoculated in casitone-yeast extract (CY) broth at 30 °C and 120 rpm in a shaking incubator (H1012 Incu-Shaker, Benchmark Scientific Inc., Edison, NJ, USA) for 3 days [31]. Then, 200 µL of the bacterial culture (10^5^ cells/mL) was inoculated again in 200 mL of CY medium containing 1% chitin powder. The experiment was conducted in three replicates and the flasks were incubated at 30 °C in a shaking incubator at 120 rpm for 10 days. Samples were collected daily for 10 days. Then, the samples were serially diluted and spread on the CYA medium. The plates were incubated at 30 °C for 3 days. Bacteria cells release extracellular metabolites such as hydrolytic enzymes during growth and thus cell growth can be used to predict the point of maximum efficacy of the bacteria culture for field application [32]. The number of cells on CYA medium were counted each incubation day to determine the growth pattern of *L. antibioticus* HS124.

### 2.3. Production of Cuticle Degrading Enzymes by Lysobacter antibioticus HS124

To examine the chitinase- and protease-producing activity of *L. antibioticus* HS124 during the incubation period, bacteria cultures were collected daily for 10 days and kept at −70 °C until analyzed. The cultures were thawed and centrifuged at 12,000 rpm for 10 min in a centrifuge machine (Combi R515, Hanil Scientific Inc., Seoul, Korea). The supernatants were used to analyze chitinase and protease activity. Chitinase activity was measured as previously described [35]. Reaction mixtures were prepared in Eppendorf tubes by mixing 50 µL of bacterial supernatant, 450 µL of 50 mM sodium acetate buffer (pH 5.0), and 500 µL of 0.5% colloidal chitin solution. The tubes were then incubated at 37 °C for 1 h. To end the reaction, 200 µL of 1 N NaOH was added and the tubes were centrifuged at 12,000 rpm for 7 min at 4 °C. Then, 750 µL of the supernatant was mixed with 1 mL of Schales’ reagent and 250 µL of distilled water and the mixture boiled 100 °C for 15 min. The amount of reducing sugar was determined using a UV spectrophotometer (UV-1650PC, Shimadzu, Kyoto, Japan) at an absorbance of 420 nm. One unit of chitinase activity was defined as the reducing activity that released 1 µmol of N-acetyl-glucosamine per h at 37 °C.

Protease activity was determined following a preexisting method [36] wherein tris buffer (100 mM) containing 2 mM CaCl_2_ and 1% casein was prepared and adjusted to pH 8.0. A reaction mixture containing 50 µL of bacterial supernatant and 950 µL of tris buffer was incubated at 60 °C for 15 min. Then, 500 µL of 20% trichloroacetic acid was added to terminate the reaction. The mixture was centrifuged at 13,000 rpm for 15 min and absorbance of the supernatant containing acid-soluble proteins was measured at 280 nm using a UV spectrophotometer. One unit of protease activity was defined as the amount of enzyme that liberated 1 µg of tyrosine per min.

### 2.4. Identification of Chitinase and Protease Genes from the Lysobacter antibioticus HS124 Genome

The bioinformatic data lays a strong foundation for obtaining in-depth knowledge about hydrolytic enzymes deployed by entomopathogenic *L. antibioticus* HS124. A local-blast database was constructed using the makeblastdb program with the *L. antibioticus* HS124 genome (CAQP01.1.fsa_nt) obtained from GenBank (https://www.ncbi.nlm.nih.gov/ (accessed on 3 July 2020)). Then, chitinase and protease genes were identified by local-tblastn analysis with *Lysobacter* chitinase and protease protein sequences obtained from GenBank (Tables S1 and S2) as a query for *L. antibioticus* HS124 genome database. Identified chitinase and protease genes were confirmed by blastp analysis (https://blast.ncbi.nlm.nih.gov/Blast.cgi (accessed on 3 July 2020)) with the nr database. Additionally, the signal peptide domain of these genes was predicted by the SignalP-5.0 program (http://www.cbs.dtu.dk/services/SignalP/ (accessed on 3 July 2020)). 

### 2.5. Preparation of Bacterial Culture and Crude Enzymes from Lysobacter antibioticus HS124

To obtain the bacterial culture and crude enzymes for assessing *L. antibioticus* HS124 larvicidal activity against the *H. cunea* larvae, the bacteria colony was inoculated into 5 L of CY medium containing 1% chitin powder and incubated at 30 °C and 120 rpm on a shaking incubator for 7 days. Then, 1 L of the bacterial culture was maintained at 4 °C for the larvicidal activity experiment. The remaining 4 L of bacterial culture was centrifuged at 6000 rpm for 30 min and the supernatant was filtered through four layers of filter paper (Whatman No.6, Whatman International Ltd., Maidstone, England). The filtered supernatant was precipitated with 80% ammonium sulfate by gently stirring at 4 °C. Then, the solution was maintained overnight at 4 °C to enhance protein precipitation and stabilization. The precipitated crude enzymes were collected from the mixture by centrifugation at 6000 rpm for 30 min and the pellet containing crude enzymes was dissolved in a small amount of 50 mM potassium phosphate buffer (pH 6.0). Then, the crude enzymes were kept in a dialysis tube and dialyzed against the same buffer at 4 °C for 24 h in a refrigerator. The total protein concentration of crude enzymes was determined using bovine serum albumin as the standard [37]. The crude enzymes were maintained at −70 °C for the larvicidal activity experiment against the *H. cunea* larvae.

### 2.6. Larvicidal Activity of Lysobacter antibioticus HS124 against the Hyphantria cunea Larvae

To examine the larvicidal activity of *L. antibioticus* HS124 against the *H. cunea* larvae, four treatments, including a control (distilled water), the CY medium, bacterial culture, and bacterial crude enzymes, were tested in the laboratory. The CY medium and bacterial culture were diluted with water (1:2 *v*/*v*) to maintain uniformity with the concentration of *L. antibioticus* HS124 cultures used in the field experiments. CY medium was used as a control for *L. antibioticus* HS124 bacteria culture, which was prepared in the same media. The CY medium application rate was based on the basal CY medium application rate for the *L. antibioticus* HS124 culture.

In July 2019, *H. cunea* larvae (0.6–0.8 cm) were collected from the leaves of *P. canadensis* from an untreated field in the study area. The larvae were maintained in an insect rearing cage (55 cm width × 35 cm length × 40 cm depth) at 25 ± 2 °C and 75 ± 10% relative humidity under natural light/dark conditions (14 h light/10 h dark). Larvae were reared under the above conditions for 1 week and fresh *P. canadensis* leaves were supplied twice. For the laboratory bioassay, *P. canadensis* leaves were collected from 2-year-old cuttings in a greenhouse with an automatic spray irrigation system at the forest nursery of the Chonnam National University. The leaves were cut to the same size (8 cm wide × 8 cm long) and cleaned with distilled water three times to remove other substances adhered to the leaves. The leaves were then air-dried at room temperature (approximately 25 °C) and one leaf was placed in each insect breeding Petri dish and 10 larvae were placed on each leaf.

For each treatment solution, 2 mL were sprayed on the larvae and leaf in each Petri dish and each treatment was conducted in three replicates. The dead larvae were counted daily for 10 days and the mortality rate (%) was calculated based on the ratio of dead larvae to total larvae. The dead larvae in each treatment were preserved in separate sterile vials containing 4% paraformaldehyde solution and kept at 4 °C for scanning electron microscopy (SEM; GeminiSEM 500, Carl Zeiss AG, Oberkochen, Germany).

### 2.7. Morphological Degradation of the Hyphantria cunea Larvae by Lysobacter antibioticus HS124

To examine the effect of *L. antibioticus* HS124 on cuticle morphology of *H. cunea* larvae, the fixed samples were washed twice with phosphate-buffered saline (pH 7.4). The samples were first dehydrated through a series of increasing alcohol concentration, i.e., 30, 50, 70, 80, 95, and 100% for 30 min each. Finally, the samples were dehydrated with isoamyl acetate for 1 h and air-dried under a fume hood overnight. The dried samples were coated with gold-nano particles at 60 °C and then observed on SEM at a magnification of 500× to investigate morphological degradation on the cuticle.

### 2.8. Indole-3-Acetic Acid Production by Lysobacter antibioticus HS124

Quantitative analysis of IAA production by *L. antibioticus* HS124 was performed using a UV spectrometric method as described previously [38]. Briefly, *L. antibioticus* HS124 was cultured in the CY medium containing L-tryptophan (0.1 g/L). The culture was incubated at 30 °C in a shaking incubator (140 rpm). Samples were taken every day for 10 days beginning on the day of inoculation. The samples were immediately centrifuged at 12,000 rpm for 10 min at 4 °C and 1 mL of the supernatant was mixed with 2 mL of Salkowski’s reagent. Subsequently, the reaction mixture was incubated at room temperature in the dark for 25 min. IAA concentration of each sample was measured at 530 nm using a UV spectrometer.

### 2.9. Field Experimental Conditions and Plant Sampling

The study was set up in two experimental groups, (1) control (without water and bacterial culture) and (2) *L. antibioticus* HS124 culture. Each group was divided into 3 plots (replicates) containing uniformly planted with *P. canadensis* trees. The *L. antibioticus* HS124 cultures were prepared in the CY medium. A liquid form of the microbial product containing 10^10^ cells/mL of *L. antibioticus* HS124 (GCM+, Purne, Jangseong, Korea) was used for large-scale cultivation of *L. antibioticus* HS124 at the field experimental site (Figure 1B(b)). Typically, 300 mL of *L. antibioticus* HS124 liquid product was inoculated into 1000 L of CY medium and cultured at 30 °C for 7 days using a fermenter (M-1000, MC Biotec, Gokseong, Korea). Then, *L. antibioticus* HS124 culture was diluted with tap water (1:2 *v*/*v*) and sprayed onto the leaves of *P. canadensis* trees. The incidence of *H. cunea* in Korea occurred from May to September 2016 and 2017. *H. cunea* is mainly bivoltine in South Korea. The eggs of the first-generation hatch in May and the last generation overwinter in September. Therefore, the treatments were applied at an interval of approximately 2–3 weeks, depending on weather conditions (carefully avoiding treatment wash-off or over-dilution by rainy weather). Therefore, a total of 5 and 6 applications of entomopathogenic *L. antibioticus* HS124 culture or water were done using a vehicle-mounted sprayer (HT-DV100, Zhengzhou Honest Machinery co., Ltd., Henan, China) from June to August in 2016 and 2017, respectively (Figure 1C). One week after every treatment application, 20 dead larvae were randomly collected from each plot and kept in 4% formaldehyde (fixating) solution to examine the morphological characteristics using SEM. Since the control group had no fatality, 20 living larvae were randomly picked and fixated for SEM morphological analysis.

To determine the effect of *L. antibioticus* HS124 culture treatment on tree growth (root collar diameter and height) and biomass production (leaf and stem dry weight), 20 trees were randomly harvested in October 2017. The diameter and lengths of shoot and root were measured using a centimeter (cm) ruler. To determine the biomass, shoots and roots of each treatment were dried for 24 h in pre-weighed, moisture-free paper bag at 105 °C, in a convection drying oven (VS-1202D4, Vision Scientific, Daejeon, Korea) and the dry weight was measured (kg) using electronic balance.

### 2.10. Statistical Analysis

Statistical analysis was conducted using statistical software (SPSS 25.0, SPSS Inc., Chicago, IL, USA). The data for cutting growth and biomass parameters were subjected to a *t*-test analysis, with significance level set at α = 0.05. The data of cell growth, cuticle-degrading enzymes production, and IAA production were subjected to an analysis of variance (ANOVA) using Waller–Duncan test with a significance level set at α = 0.05. The larvae mortality data analyzed by two-way ANOVA, with the treatments and time post-application as factors at α = 0.05. The results are reported as the mean ± standard errors.

## 3. Results

### 3.1. Larvicidal Activity of Lysobacter antibioticus HS124 on the Hyphantria cunea Larvae

#### 3.1.1. Growth Pattern of *Lysobacter antibioticus* HS124

**Figure 2:**
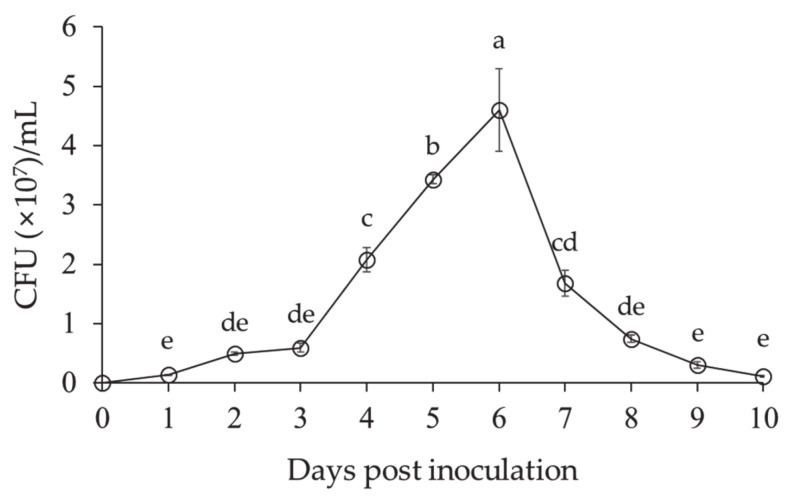
Cell growth curve of *Lysobacter antibioticus* HS124 in CY medium. Values are represented as means ± standard errors (*n* = 3). Different superscripts in the figure indicate significantly different values using Waller–Duncan test (*p* < 0.05).

The growth of *L. antibioticus* HS124 was low until 3 days after inoculation (Figure 2). Thereafter, growth of *L. antibioticus* HS124 rapidly increased until 6 days after inoculation, when the growth rate reached a maximum value of 4.6 × 10^7^ cell/mL. The growth of *L. antibioticus* HS124 sharply declined from 7 days post-inoculation until the end of the experimental period (Figure 2). 

#### 3.1.2. Cuticle Degrading Enzymes Production by *Lysobacter antibioticus* HS124

**Figure 3:**
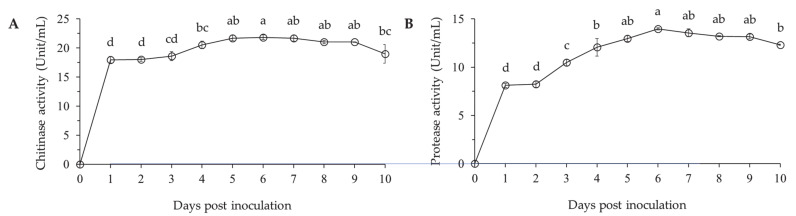
Chitinase (**A**) and protease (**B**) activity by *Lysobacter antibioticus* HS124 in CY medium. Values are represented as means ± standard errors (*n* = 3). Different superscripts in the figure indicate significantly different values using Waller–Duncan test (*p* < 0.05).

The chitinase activity of *L. antibioticus* HS124 was stable until 3 days after inoculation (Figure 3A). Thereafter, enzyme activity gradually increased from 4 days after inoculation and remained steady until 5 days after inoculation. The highest enzymatic activity was observed at 6 days after inoculation with a value of 21.85 unit/mL. A slight decrease in chitinase enzyme activity was observed at the final day of the incubation. 

The protease activity of *L. antibioticus* HS124 was stable in the first 2 days after inoculation and rapidly increased at 3 days after inoculation (Figure 3B). A continuous increase of protease activity was observed until 6 days after inoculation, reaching a maximum value of 13.99 unit/mL. Subsequently, the activity decreased until the end of the incubation period. 

Based on the bioinformatic analysis, 6 chitinase (or chitinase-like) and 29 protease genes were identified from *L. antibioticus* HS124 (Tables S1 and S2). The identified protease genes included 6 metalloproteases, 1 signal peptide peptidase, 4 ATP-dependent protease, 8 protease, and 10 serine protease genes. Interestingly, four of six chitinase genes included the signal peptide domain. Moreover, protease genes, which included the signal peptide domain, were mainly involved in the protease and serine protease groups.

#### 3.1.3. *Hyphantria cunea* Larvae Mortality of *Lysobacter antibioticus* HS124

**Figure 4:**
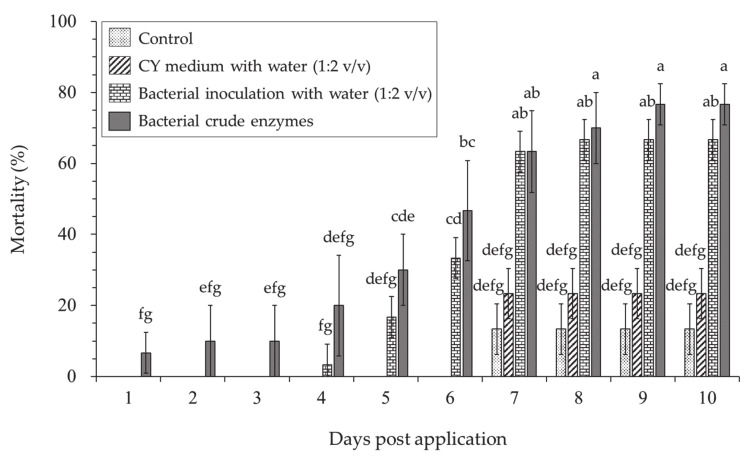
Average mortality rate of *Hyphantria cunea* larvae in control (distilled water), CY medium diluted with distilled water (1:2 *v*/*v*), bacterial culture diluted with distilled water (1:2 *v*/*v*), and crude enzymes from *Lysobacter antibioticus* HS124 treatment in laboratory experiment. Error bars represent standard errors of the mean (*n* = 3) for each day. Means were separated by Waller–Duncan multiple range test. Means with different superscripts in the figure indicate significantly different values.

Treatment with crude enzymes and *L. antibioticus* HS124 bacteria culture with water (1:2 *v*/*v*) were highly effective to induce *H. cunea* larvae mortality in the laboratory (Figure 4). Treatment with crude enzymes showed relatively higher larvicidal activity against the *H. cunea* larvae, with up to 10.0% mortality, 2 days after application compared to bacteria culture treatment, which required longer time (4 days post-application) to induce mortality. The larvicidal effect of these crude enzymes increased with time (post-application days) and the highest mortality rate (76.6%) was observed 9 days after treatment (Figure 4). Only 16.7% larvae mortality rate was observed in the bacteria culture treatment after 5 days, but the rate increased 4 times to 66.7% 8 days post-application. The time post-application had a significant effect on larvae mortality. In overall, the larvicidal activity of crude enzymes and bacteria culture were observed from 1 to 9 days and 4 to 8 days post-application, respectively. Crude enzyme treatment showed significantly higher efficacy than the bacteria culture (Figure 4). Moreover, treatment and post-application time interaction had a significant effect on larvae mortality. No significant difference was observed between control (water) and CY media and larvae mortality in both groups were only observed 7 days post-application. The maximum larvae mortality of 23.3 % and 13.3 % were observed in CY medium with water (1:2 *v*/*v*) and control (water) at 10 days post-application, respectively (Figure 4).

Foliar application of *L. antibioticus* HS124 culture on *P. canadensis* trees in the field experiment was conducted in an attempt to control the *H. cunea* larvae (Figure 1C(d–e)). *P. canadensis* trees treated with the bacterial culture little damage caused by the larvae on the leaves (Figure 1C(d)). Additionally, *H. cunea* larvae activity was rarely observed in the bacterial treatment group. However, in the control field, *H. cunea* larvae were spread throughout the leaves of the *P. canadensis* trees and innumerable webbings and larva exuviae formed on the trees as shown (Figure 1C(a–b)). The larvae feeding activity resulted in severe damage on *P. canadensis* trees, causing almost total defoliation as shown (Figure 1C(c)).

#### 3.1.4. Morphological Deformation of the *Hyphantria cunea* Larvae

**Figure 5:**
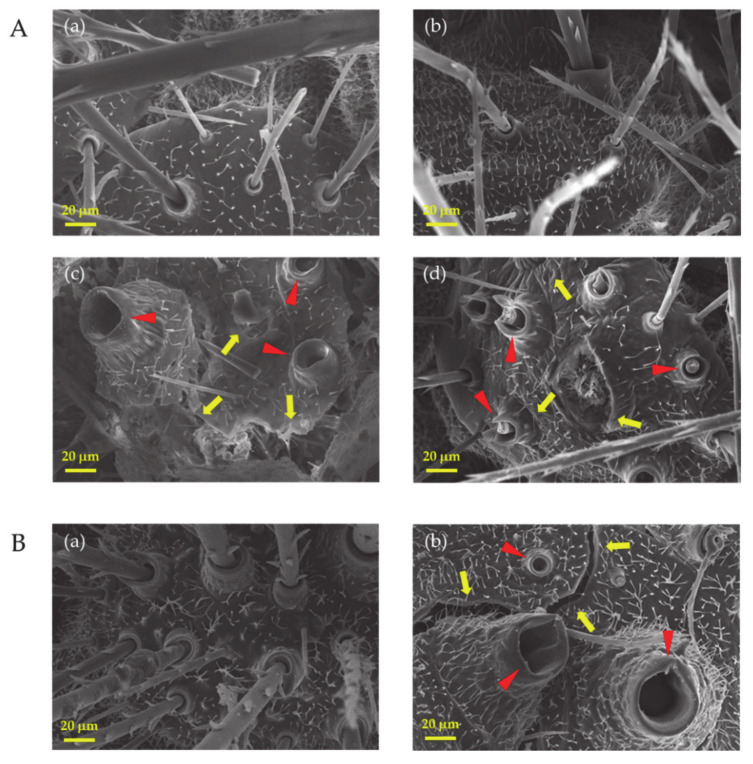
Exoskeletons of *Hyphantria cunea* larvae observed using SEM, the control (distilled water) (**A**a), CY media diluted with distilled water (1:2 *v*/*v*) (**A**b), bacterial culture diluted with distilled water (1:2 *v*/*v*) (**c**), and crude enzymes from *Lysobacter antibioticus* HS124 treatment (**d**) from the laboratory experiment (**A**). Normal structural in the control group (**B**a) and bacterial culture diluted with tap water (1:2 *v*/*v*) (**B**b) from the field experiment (**B**). Yellow arrows indicate the rupture of exoskeletons and red arrows indicate swollen sockets with damaged setae.

SEM analysis of dead larvae from the laboratory experiment revealed differences between treatments and control group (Figure 5A). The larvae cuticle of the dead larvae in the control (Figure 5A(a)) and CY media (Figure 5A(b)) showed normal morphologies without disruption or damage to the cuticle and setae. However, the morphological features of dead larvae from both the bacterial culture (Figure 5A(c)) and crude enzymes (Figure 5A(d)), showed abnormal morphologies, characterized by substantial loss of setae from the cuticles (indicated by the red arrows) and swelling of sockets at the base of setae, leading to complete rupture of the exoskeleton (yellow arrows).

Similarly, the cuticle of dead larvae collected from the field experiment displayed substantial differences between the control (Figure 5B(a)) and bacterial treatment (Figure 5B(b)). The larvae cuticles in the treatment group showed broken setae (red arrows), with swollen sockets and ruptured cuticles (yellow arrows) while the control group displayed normal structure with setae and healthy cuticles.

### 3.2. Promotion Effects of Lysobacter antibioticus HS124 on Growth of Populus canadensis

#### 3.2.1. Indole-3-Acetic Acid Production of *Lysobacter antibioticus* HS124

**Figure 6:**
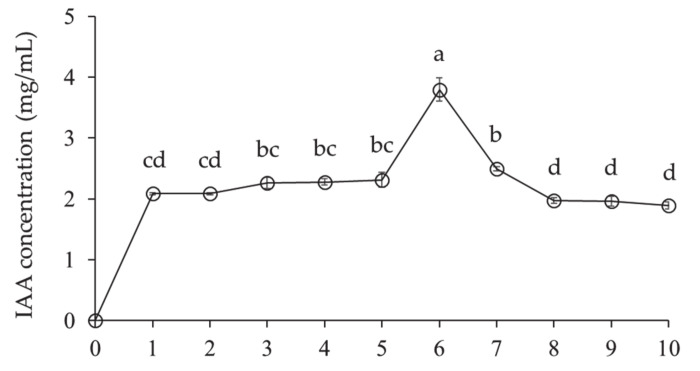
Indole-3-acetic acid production by *Lysobacter antibioticus* HS124. Values are represented as means ± standard errors (*n* = 3). Different superscripts in the figure indicate significantly different values using Waller–Duncan test (*p* < 0.05).

*Lysobacter antibioticus* HS124 produced auxin, IAA during growth (Figure 6). The IAA concentration steadily increased for 5 days, eventually reaching a maximum value of 3.8 mg/mL in 6 days. Thereafter, the IAA concentration decreased gradually.

#### 3.2.2. Growth and Biomass Yield of *Populus canadensis* Trees

A significant increase in growth (root collar diameter and height) and biomass yield (leaf and stem dry weight) was observed in *P. canadensis* trees treated with the *L. antibioticus* HS124 culture compared to the control group (Table 1). When compared to the control group, treatment with *L. antibioticus* HS124 increased root collar diameter and tree height by 1.7- and 1.4-fold, respectively. Strikingly, the stem dry weight of the treatment group was 5.1-fold higher than that of the control group (Table 1). However, there were no leaves on *P. canadensis* trees in the control group due to severe defoliation under natural infestation by *H. cunea* larvae at the field (Table 1).

## 4. Discussion

### 4.1. Larvicidal Activity of Lysobacter antibioticus HS124 against the Hyphantria cunea Larvae

The cuticle-degrading chitinases and proteases used by entomopathogenic bacteria to penetrate insect structural barriers can be potentially useful in insect pest management. These enzymes weaken the structural defense system and thus increase the vulnerability of insect to external aggressions [20,39]. In the present study, *L. antibioticus* HS124 secreted cuticle-degrading chitinase and protease (Figure 3). Bioinformatic analysis indicated that there are 6 and 29 putatively secreting genes for chitinase and proteases, respectively (Tables S1 and S2). Especially, several chitinase glycoside hydrolase subfamily 18 (GH18) genes and serine protease genes were identified from *L. antibioticus* HS124 (Tables S1 and S2). GH18 chitinase is responsible for chitinase enzyme production, which then degrades chitin into low molecular weight of N-acetylglucosamine such as chitotetraose, chitotriose, and chitobiose [27,29]. This leads to structural disintegration of vital insect organs such as the cuticle [28,40], causing mortality of insect pests due to the hydrolytic activity in the larvae cuticles [28,41,42]. In addition, serine proteases have extensive primary specificity for amino acids (e.g., phenylalanine, methionine, and alanine) with a hydrophobic side group in the second carbon atom, but also has a secondary specificity for extended peptide chains with active sites recognizing at least five subsite residues [29]. Due to this comparative non-specificity, the general protease could have high hydrolytic activity on the insect exoskeleton by breaking down various proteins (casein, elastin, bovine serum albumin, and collagen) present in the larval cuticle [25,29]. These genes have indicated that *L. antibioticus* HS124 causes hydrolytic activity in the larvae cuticle and could potentially play an important role in insect pest management.

In laboratory experiments, the highest insecticidal activity of *L. antibioticus* HS124 crude enzyme and bacteria culture against *H. cunea* larvae was 76.6% and 66.7% at 10 days post application, respectively (Figure 4). Crude enzyme treatment showed higher efficacy, causing early mortality compared to bacterial culture (Figure 4). The slightly lower effect of bacteria culture compared to crude enzyme could be related to culture dilution (1:2 *v*/*v* with water). The larvae mortality was consistent with the rupturing of the outermost epicuticle (Figure 5A) which could be a result of increased stress caused by hydrolytic activity of cuticle-degrading enzymes. Treatment with *L. antibioticus* HS124 culture or crude enzymes caused a drastic morphological deformation of *H. cunea* larvae cuticles, causing swelling and rupturing of the exoskeleton (Figure 5A). Additionally, there was a pronounced loss of larval socketed setae (Figure 5A). The loss of socketed setae of *H. cumea* caused more stress, due to the physical impact on the epicuticle and water loss [24]. On the other hand, the mortality observed in the CY medium and control was only 23.3% and 13.3% after 10 days of treatment, respectively. Moreover, the cuticles of dead larvae in the CY media and control showed the normal shape of the larval socketed setae and cuticles. The disintegration of the larvae cuticle, deformation of socketed setae, and the subsequent larvae mortality in insects treated with a bacterial culture or crude enzyme suggests that the entomopathogenic bacterium *L. antibioticus* HS124 could be an effective biopesticide against *H. cumea*. Foliar application of *L. antibioticus* HS124 culture using a vehicle-mounted sprayer machine on *P. canadensis* trees in the field experiment resulted in a rapid decrease in larvae density and subsequent reduction in the level of leaf damage caused by *H. cumea* (Figure 1). Similarly, the cuticle of dead larvae in field experiments were also observed, and the larvae treated with bacterial cultures showed cuticle disruption and deformation of socketed setae (Figure 5B). In contrast, the cuticle of the dead larvae in the control group was found to have a normal morphology without degradation damage (Figure 5B). The activity of chitinase and protease from entomopathogenic bacteria against pests has been well documented at laboratory scale [20,21]. However, the efficacy of forest-scale application of bacterial culture containing cuticle-degrading enzymes, such as chitinase and protease, secreted by entomopathogenic bacteria remains unknown. Based on molecular and biochemical results of this study, the entomopathogenic bacterium *L. antibioticus* HS124 could be adopted as a biopesticide to control *H. cunea* on a large-scale forest setting.

### 4.2. Promotion Effects of Lysobacter antibioticus HS124 on Growth of Populus canadensis

The application of *L. antibioticus* HS124 culture resulted in significant growth promotion of *P. canadensis* trees through the control of pest activity and enhancement of the growth via phytohormones such as auxin. The *P. canadensis* cuttings in the control group grew to average of 4.0 cm in root collar diameter and 444.4 cm in height (Table 1). With the foliar application of *L. antibioticus* HS124 culture, the root collar diameter and height of *P. canadensis* cuttings increased by 1.7- and 1.4-fold, respectively (Table 1). Compared to the total biomass (0.9 kg) in the control group, the *P. canadensis* cuttings treated with bacterial culture had a 5.4-fold increase in total biomass yield (Table 1). Since 2015, the *P. canadensis* plantations have suffered widespread defoliation due to *H. cunea* infestation, resulting in a serious decrease in biomass yields (Figure 1). Leaves are the major determinants of biomass productivity [43,44]. They play a crucial role in photosynthesis, thereby providing carbohydrates for other plant metabolic processes. Loss of leaf area reduces plant surface area for light absorption, which can significantly affect the photosynthetic rate and in turn reduce tree growth and biomass production [43]. Moreover, photosynthesis also plays a vital role in carbon sequestration, which is an important aspect of forest ecosystem and climate mitigation [43]. In this study, *P. canadensis* cuttings treated with *L. antibioticus* HS124 culture provided sufficient protection against the destructive phytophagous *H. cunea* larvae via cuticle-degrading enzymes such as chitinase and protease (Figure 1 and Figure 3). Consequently, treatment with *L. antibioticus* HS124 prevented tree defoliation, thereby guaranteeing an adequate leaf surface and thus contributing to the high photosynthetic activity compared to the control group.

In addition, *L. antibioticus* HS124 produced the IAA phytohormone (Figure 6), which acts as a general coordinator of plant growth and development [7,8,25]. IAA is absorbed into leaves through foliar application, promoting biomass production through rapid growth such as shoot tissue emergence, shoot length extension, and young leaves development [31,45]. Then, IAA moves cell-to-cell transports from shoots to roots by polar auxin transport [45]. IAA enters plant cells by two interconnected transport systems: (1) the non-ionized protonated indole-3-acetamide hydrolase (IAAH), which is lipophilic and therefore enters the plant cell through the lipid bilayer by passive diffusion, and (2) anionic form IAA^-^, which enters into the cytoplasm by active co-transport by auxin influx carriers such as auxin-resistant/like aux (AUX/LAX) present in the cell membrane [46,47]. Once through the lipid bilayer into the cells, the molecules are exposed to a more basic pH within the cells and are almost completely dissociated to produce anionic IAA, which crosses the lipid bilayer to leave the cells [46,47]. Hence, IAA can be transported to the cell wall space only by the auxin efflux carriers, an active transport component in the plasma membrane [45,46,47]. Among the auxin efflux carriers, the downward transportation from shoot to the root occurs by pin-formed (PIN) proteins located only in the basement membrane (i.e., on their lower side) [45,46]. The IAA that reaches the roots causes root hair development and lateral root formation, thereby increasing the surface area of the root system in contact with the soil [33]. As a result, trees can absorb more water and mineral nutrients, thereby improving plant growth and biomass production [33]. Therefore, foliar application of IAA can greatly improve growth and biomass production in seedlings [30], which is also consistent with the results of the current study. In addition, exogenous auxin treatment triggers increased levels of endogenous auxin, thereby activating cambial growth, and the internal auxin acts as a growth regulator for the entire plant [30]. Therefore, *L. antibioticus* HS124 treatment increased plant growth and biomass of *P. canandensis* compared to the control (Table 1) due to IAA production (Figure 6).

## 5. Conclusions

Formulations of chitinases and proteases with other biopesticides or chemically synthesized pesticides might allow for a reduction in the environmental impact of toxic chemical compounds and reduce the risk of pesticide resistance. In addition, auxin improved the growth of poplar trees by promoting cell division, tissue growth, and lateral root initiation. In conclusion, this work sheds light on (i) the efficacy of investigations into biological pesticide development and use of biocontrol technologies as part of integrated pest management programs, and (ii) the need to develop unconventional heterologous platforms to initiate biomass production in large-scale forests. Microbial technology is crucial to the development of novel pesticides and sustainable biomass production. To the best of our knowledge, this is the first report on a forest-scale demonstration of simultaneous improvement in forest biomass production by controlling of forest pests with cuticle degrading enzymes such as chitinase and protease secreted by entomopathogenic bacteria and promotion of tree growth by phytohormone production.

## Figures and Tables

**Table 1:** Growth and biomass production of *Populus canadensis* for the control vs *Lysobacter antibioticus* HS124 culture treatment in a coastal reclaimed land.

Treatment	Tree Growth (cm)		Tree Biomass (kg)		
	Root Collar Diameter	Height	Leaf Dry Weight	Stem Dry Weight	Total
Control	4.0 ± 0.1 *	444.4 ± 13.0 *	0.0 ± 0.0 *	0.9 ± 0.1 *	0.9 ± 0.1 *
*Lysobacterantibioticus* HS124	6.7 ± 0.2 *	604.4 ± 21.9 *	0.4 ± 0.0 *	4.5 ± 0.3 *	4.9 ± 0.3 *
*p*-value	0.001	0.004	<0.001	<0.001	<0.001

Values are means ± standard errors. * Indicates a significant difference between means in the same column.

## Data Availability

Data available on request from the corresponding author.

## Supplementary Materials

The following are available online at https://www.mdpi.com/article/10.3390/microorganisms9081580/s1, Table S1: List of chitinase genes identified in the *L. antibioticus* HS124 genome. Table S2: List of protease genes identified in the *L. antibioticus* HS124 genome.
