# Supplementary material for: The Role of Lysobacter antibioticus HS124 on the Control of Fall Webworm (Hyphantria cunea Drury) and Growth Promotion of Canadian Poplar (Populus canadensis Moench) at Saemangeum Reclaimed Land in Korea
Source: Microorganisms. 2021 Jul 25;9(8):1580. doi: 10.3390/microorganisms9081580 (PMC8398145; doi:10.3390/microorganisms9081580)
Supplement: Supplementary file 1 [file microorganisms-09-01580-s001.zip › Supplementary.pdf]

**Supplementary Material**

**Table S1.** List of chitinase genes identified in the *L. antibioticus* HS124 genome.

| *L. antibioticus* HS124 Accession No. | Initiation codon | Termination codon | NT length | AA length | Annotation | DB Accession No. | Signal peptide |
|---|---|---|---|---|---|---|---|
| CAQP01000049.1 | 3570 | 1801 | 1770 | 589 | GH18* chitinase-like | WP_079248128.1 | SMA-AE |
| CAQP01000049.1 | 18870 | 20834 | 1965 | 654 | fibronectin type III domain-containing protein chitinase | WP_079248132.1 ALN55759.1 | - |
| CAQP01000080.1 | 8514 | 10568 | 2055 | 684 | fibronectin type III domain-containing protein chitinase A | WP_079248209.1 AAT77163.1 | AYA-QA |
| CAQP01000109.1 | 22976 | 23848 | 873 | 290 | GH18* chitinase | ALN82488.1 | CAA-RN |
| CAQP01000196.1 | 1019 | 1636 | 618 | 205 | chitinase class I family protein | ALN85183.1 | - |
| CAQP01000222.1 | 3082 | 1367 | 1716 | 571 | GH18* chitinase-like | WP_079248394.1 | AFG-MD |

* GH18; glycosyl hydrolases 18 family protein.

**Table S2**. List of protease genes identified in the *L. antibioticus* HS124 genome.

| *L. antibioticus* HS124 Accession No. | Initiation codon | Termination codon | NT length | AA length | Annotation | DB Accession No. | Signal peptide |
|---|---|---|---|---|---|---|---|
| CAQP01000010.1 | 24236 | 26152 | 1917 | 638 | M48 family metalloprotease | WP_079247980.1 | |
| CAQP01000011.1 | 17644 | 19410 | 1767 | 588 | M48 family metallopeptidase | WP_079247985.1 | |
| CAQP01000031.1 | 30109 | 32061 | 1953 | 650 | metalloendopeptidase CpaA | WP_079248080.1 | |
| CAQP01000089.1 | 2574 | 673 | 1902 | 633 | ATP-dependent zinc metalloprotease FtsH | WP_064748602.1 | FQA-FG |
| CAQP01000089.1 | 29400 | 30998 | 1599 | 532 | M4 family metallopeptidase | WP_075575174.1 | |
| CAQP01000096.1 | 40826 | 39465 | 1362 | 453 | RIP metalloprotease RseP | WP_064748791.1 | |
| CAQP01000015.1 | 6867 | 4966 | 1902 | 633 | signal peptide peptidase SppA | WP_064746495.1 | |
| CAQP01000021.1 | 8545 | 6263 | 2283 | 760 | ATP-dependent Clp protease ATP-binding subunit ClpA | WP_064746694.1 | |
| CAQP01000021.1 | 8957 | 8637 | 321 | 106 | ATP-dependent Clp protease adapter ClpS | WP_064746695.1 | |

| | | | | | | | |
|---|---|---|---|---|---|---|---|
| CAQP01000056.1 | 48741 | 48298 | 444 | 147 | ATP-dependent zinc protease | WP_064747762.1 | |
| CAQP01000170.1 | 9692 | 10243 | 552 | 183 | ATP-dependent protease subunit HslV | WP_064749737.1 | |
| CAQP01000023.1 | 16574 | 17218 | 645 | 214 | HK97 family phage prohead protease | WP_064746799.1 | |
| CAQP01000023.1 | 61853 | 62767 | 915 | 304 | protease HtpX | WP_064746850.1 | |
| CAQP01000047.1 | 50601 | 49495 | 1107 | 368 | protease | WP_064747554.1 | AIA-AA |
| CAQP01000053.1 | 37642 | 35945 | 1698 | 565 | M28 family peptidase | WP_079248140.1 | AVA-AK |
| CAQP01000065.1 | 25742 | 24243 | 1500 | 499 | S41 family peptidase | WP_087960714.1 | GLA-RE |
| CAQP01000093.1 | 45735 | 44503 | 1233 | 410 | S1 family peptidase | WP_079248243.1 | |
| CAQP01000180.1 | 13518 | 14609 | 1092 | 363 | protease | WP_064749835.1 | VTA-AP |
| CAQP01000209.1 | 3505 | 4893 | 1389 | 462 | S41 family peptidase | WP_087960776.1 | AQA-SA |
| CAQP01000047.1 | 8657 | 9106 | 450 | 149 | rhomboid family intramembrane serine protease | WP_064747527.1 | |
| CAQP01000065.1 | 22937 | 24301 | 1365 | 454 | rhomboid family intramembrane serine protease | WP_064747982.1 | AWA-QD |
| CAQP01000077.1 | 22840 | 21179 | 1662 | 553 | S8 family serine peptidase | WP_079248202.1 | |
| CAQP01000082.1 | 119866 | 120582 | 717 | 238 | rhomboid family intramembrane serine protease | WP_064748438.1 | |
| CAQP01000085.1 | 16137 | 15355 | 783 | 260 | rhomboid family intramembrane serine protease | WP_064748495.1 | TAP-LP |
| CAQP01000093.1 | 65977 | 64409 | 1569 | 522 | Do family serine endopeptidase | WP_079248247.1 | CTA-QA |
| CAQP01000095.1 | 2978 | 1542 | 1437 | 478 | Do family serine endopeptidase | WP_064748760.1 | GFA-AT |
| CAQP01000150.1 | 27017 | 26019 | 999 | 332 | trypsin-like serine protease | WP_064749580.1 | AEA-KE |
| CAQP01000157.1 | 1381 | 500 | 882 | 293 | trypsin-like serine protease | WP_064749632.1 | AGA-VV |
| CAQP01000199.1 | 27984 | 29525 | 1542 | 513 | trypsin-like serine protease | WP_141233619.1 | LQA-QP |
